# Enhanced Piezoelectric Performance of Highly-Aligned ZnO Nanorods Embedded in P(VDF-TrFE) Nanofiber Membranes

**DOI:** 10.3390/polym17050585

**Published:** 2025-02-22

**Authors:** Xingjia Li, Zhongbo Zhang, Jianjun Ye, Yuan Li, Qichao Li, Han Wang, Xiuli Zhang, Yiping Guo

**Affiliations:** 1 School of Mathematics, Physics and Statistics, Shanghai University of Engineering Science, Shanghai 201620, China; xjli@sues.edu.cn (X.L.); jjye@sues.edu.cn (J.Y.); 2 School of Chemistry and Chemical Engineering, Shanghai University of Engineering Science, Shanghai 201620, China; zhangzhongbo@sues.edu.cn (Z.Z.); wanghan@sues.edu.cn (H.W.); 3 State Key Laboratory of Metal Matrix Composites, School of Materials Science and Engineering, Shanghai Jiao Tong University, Shanghai 200240, China; yuan.li@sjtu.edu.cn (Y.L.); liqichao@sjtu.edu.cn (Q.L.)

**Keywords:** P(VDF-TrFE)/ZnO nanocomposite, P(VDF-TrFE) nanofibers, ZnO nanorods, piezoelectricity, flexible electromechanical sensors

## Abstract

Flexible and wearable electronics often rely on piezoelectric materials, and Poly(vinylidene fluoride-trifluoroethylene) (P(VDF-TrFE)) membranes are popular for this application. However, their electromechanical performance is limited due to a relatively low piezoelectric coefficient. To address this, this study investigates the incorporation of zinc oxide (ZnO) nanorods (NRs) into a P(VDF-TrFE) nanofiber membrane matrix. ZnO NRs were synthesized and doped into well-aligned P(VDF-TrFE) nanofibers using electrospinning with a high-speed rotating drum. The impact of ZnO NRs’ mass fraction on the piezoelectric properties of the membranes was evaluated. Results show that a maximum piezoelectric coefficient ($d_{33}$) of −62.4 pC/N, 9.5 times higher than neat P(VDF-TrFE), was achieved. These enhanced membranes demonstrated excellent performance in finger-tapping and bending detection, making them promising for large-scale flexible sensor applications in wearable electronics. This approach offers a simple and effective route to improve the performance of piezoelectric materials in flexible devices.

## 1. Introduction

Rapid advancements in science and technology have increased the demand for portable and wearable electronic devices. These devices are used in diverse sectors, including healthcare [1], motion monitoring [2], environmental monitoring [3], and data transmission and processing [4]. Traditional battery-based power sources have several limitations. These include their large size, short lifespan, limited integration capabilities, environmental impact, and poor biocompatibility [5]. These challenges underscore the need for alternative, clean, and sustainable power solutions that are portable and flexible. Many wearable electronics have low energy consumption. As a result, harvesting energy from the environment—such as solar, thermal, and mechanical sources—presents a promising solution [6]. Among the various energy harvesting technologies developed, including photovoltaics [7], thermoelectrics [8], magnetoelectrics [9], piezoelectric nanogenerators (PENGs) [10], and frictional nanogenerators [11], PENGs stand out for their ability to provide flexible, lightweight, and sustainable power [12]. These advantages make PENGs ideal for a wide range of applications, including electronic skin [13], healthcare [14], gesture recognition [15], and artificial synapses [16].

Piezoelectric materials are a class of materials capable of converting mechanical energy into electrical energy. These materials are broadly categorized into inorganic piezoelectric materials and piezoelectric polymers [17,18,19,20,21]. Inorganic piezoelectric materials typically exhibit higher electrical activity compared to polymers, offering greater sensitivity to external mechanical stimuli and the ability to generate higher power density [22,23]. However, their intrinsic brittleness and lack of flexibility limit their applicability, particularly in flexible electronic devices. In contrast, poly(vinylidene fluoride) (PVDF) and its copolymer, poly(vinylidene fluoride-trifluoroethylene) (P(VDF-TrFE)), have been widely employed in PENGs, especially when integrated into small-scale electronics. This is due to their flexibility, biocompatibility, chemical resistance, environmental friendliness, and ease of fabrication [24,25,26,27,28].

The piezoelectric performance of P(VDF-TrFE) is closely related to its crystalline structure, which varies based on the orientation of the fluorine atoms along the polymer backbone. The material can crystallize into different phases, such as the trans–gauche–trans–gauche (TGTG) configuration (α-phase) and the all-trans (TTTT) configuration (β-phase). Among these, the β-phase exhibits the highest piezoelectric performance and is the primary contributor to P(VDF-TrFE)’s piezoelectricity [29,30]. Compared to PVDF homopolymers, P(VDF-TrFE) copolymers have a stronger tendency to crystallize into the β-phase. This is attributed to the steric effects of the third fluorine atom in the TrFE monomer units. These effects inhibit the formation of the TGT conformation [31]. These unique structural properties make P(VDF-TrFE) a promising material for flexible piezoelectric applications.

To further improve the piezoelectric properties of P(VDF-TrFE) copolymers, a commonly employed strategy is the introduction of an additional phase, such as lead zirconate titanate (PZT) [32], barium titanate (BTO) [33], graphene, metal electrodes [34], or zinc oxide (ZnO) [34,35,36]. These additives can significantly enhance the electrical response of the polymer matrix [37]. Among these materials, ZnO is a notable lead-free piezoelectric material. It is known for its high thermal stability, favorable piezoelectric and dielectric properties, and environmental compatibility [38,39,40,41].

Electrospinning offers several distinct advantages over conventional methods for fabricating flexible sensor membranes, particularly in the context of nanomaterial-based sensors. One of the primary benefits is its ability to produce highly flexible membranes with fine, continuous nanofibers that are not achievable with traditional fabrication techniques. The nanoscale fibers produced via electrospinning exhibit excellent flexibility, allowing the sensor membranes to conform to complex surfaces, which is essential for wearable or flexible sensor applications.

Another key advantage of electrospinning is its ability to generate membranes with a high surface area-to-volume ratio. This large surface area enhances the sensor’s sensitivity and responsiveness to target analytes, improving detection limits and allowing for more accurate measurements. This is particularly important for flexible sensors, where high sensitivity is required to detect small changes in the surrounding environment.

Moreover, electrospinning allows for precise control over the morphology of the fibers, including their diameter, alignment, and porosity. This level of control is crucial for optimizing the mechanical and electrical properties of the sensor membrane. The flexibility and tensile strength of electrospun fibers can be tailored to ensure that the membranes remain functional under various mechanical deformations, such as bending or stretching, which is often required in flexible electronics and wearable sensor applications.

Electrospinning also offers the capability to incorporate a wide variety of functional materials into the nanofibers, such as conductive polymers, metal nanoparticles, or biomolecules. This versatility allows the creation of multifunctional sensor membranes with enhanced properties, such as increased electrical conductivity, catalytic activity, or specific interaction with analytes, which can further improve sensor performance in diverse sensing environments.

In this study, we employ a high-speed electrospinning technique to fabricate highly aligned P(VDF-TrFE) nanofibers doped with oriented ZnO NRs. Due to their high aspect ratio, the ZnO NRs align along the direction of the P(VDF-TrFE) nanofibers during the electrospinning process. This dual alignment of nanofibers and ZnO NRs enhances the piezoelectric properties of the resulting nanocomposite membranes, which is crucial for the advancement of efficient nanogenerators. Moreover, we investigate the expanded potential applications of these optimized materials in sensing technologies. Notably, the membranes exhibit the capability to detect finger bending and tapping, demonstrating their high sensitivity and versatility. These results lay a solid foundation for the development of advanced energy harvesting systems and flexible sensing devices, underscoring the potential of this approach for next-generation wearable and self-powered electronics.

## 2. Materials and Methods

### 2.1. Materials

The 70/30 P(VDF-TrFE) powders were obtained from Kunshan Hisense Electronics, Suzhou, China. Zinc acetate dihydrate [Zn(CH_3_COO)_2_∙2H_2_O] was supplied by Shanghai Macklin Biochemical Co., Ltd., Shanghai, China. N,N-dimethylformamide (DMF), acetone, sodium hydroxide (NaOH), and ethanol were purchased from Shanghai Reagents Co., Ltd., Shanghai, China. All chemicals and materials were used as received without any further purification.

### 2.2. Methods

#### 2.2.1. Synthesis of ZnO NRs

**Figure 1:**
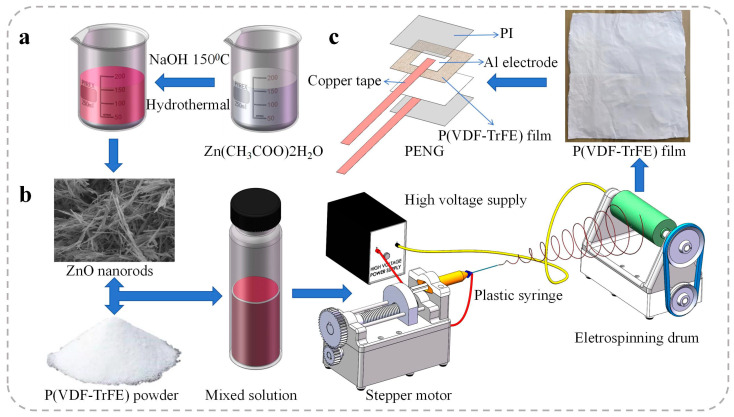
Schematic illustrations of the fabrication processes: (**a**) Synthesis of ZnO NRs using the hydrothermal method. (**b**) Fabrication of P(VDF-TrFE) fibers via the electrospinning method. (**c**) Assembly process of PENGs.

Zinc oxide nanorods (ZnO NRs) were synthesized using a hydrothermal method, as illustrated in Figure 1a. Initially, 0.88 g of Zn(CH_3_COO)_2_∙2H_2_O was dissolved in 40 mL of ethanol at a concentration of 0.1 mol/L. Separately, 3.2 g of NaOH was dissolved in 80 mL of ethanol at a concentration of 1 mol/L. Both solutions were stirred at room temperature for 2.5 h. Subsequently, the two solutions were mixed and stirred continuously for an additional 3 h. The resulting mixture was then transferred into a 250 mL Teflon-lined autoclave, which was tightly sealed and placed in an oven for hydrothermal treatment at 145 °C for 24 h. After the reaction, the autoclave was allowed to cool to room temperature naturally. The solution was then filtered and washed several times with deionized water and ethanol to remove impurities. Finally, the ZnO powder was obtained by drying the product in an oven at 75 °C for 12 h.

#### 2.2.2. Fabrication of ZnO/P(VDF-TrFE) Nanocomposite Films

The ZnO/P(VDF-TrFE) film was fabricated using an electrospinning method, as illustrated in Figure 1b. First, an appropriate amount of ZnO NRs was dispersed in 10 mL of a mixed solvent of DMF and acetone (60:40 *w*/*w*). The suspension was stirred for 30 min and subsequently subjected to ultrasonic treatment for an additional 30 min. Following this, 1.6 g of P(VDF-TrFE) was added to the ZnO suspension, and the mixture was vigorously stirred at 40 °C for 24 h until a uniform and transparent solution was obtained.

For the electrospinning process, as shown in Figure 1b, the prepared solution was loaded into a 5 mL plastic syringe equipped with a 24 G needle. The electrospinning parameters were set as follows: the applied voltage was 20 kV, the distance between the needle tip and the collector was 20 cm, and the solution flow rate was 1.32 mL/h. A rotating drum covered with aluminum foil was used as the collector, with a rotation speed of 1500 rpm to ensure fiber alignment. The temperature and relative humidity during electrospinning were maintained at 24 °C and 50%, respectively.

After electrospinning, the collected films were dried overnight at 70 °C to remove residual organic solvents. Finally, the dried membranes were annealed in a drying oven at 140 °C for 6 h and then cooled to room temperature to complete the fabrication process.

#### 2.2.3. Fabrication of PENGs

A photograph and schematic diagram of a typical nanofiber membrane device are presented in Figure 1c. The complete device, with an effective electrode area of 4 cm^2^, consists of the following layers arranged from top to bottom: a PI encapsulation layer, a top copper tape, an aluminum (Al) top electrode, the nanofiber membrane (the thickness of all the samples is 20 μm), an Al bottom electrode, a bottom copper tape, and a PI substrate.

#### 2.2.4. Characterizations

The morphologies of the ZnO NRs and ZnO/P(VDF-TrFE) films were characterized using a scanning electron microscope (SEM, model S4700, Hitachi, Tokyo, Japan). The crystal structures were analyzed by X-ray diffraction (XRD, model D/max-2200/PC, Rigaku, Wako, Japan) diffractometer with Cu Kα radiation (λ = 0.154056 nm). XRD patterns were recorded in the 2θ range of 5° to 75° with a scanning rate of 3°/min at room temperature. Fourier transform infrared (FTIR) spectra were collected using a Microscopic Infrared Spectrometer (FTIR, model 760, Nicolet, Rhinelander, WI, USA) in the wavenumber range of 4000–500 cm⁻^1^. The length and diameter distributions of the nanofibers were analyzed using the Nano-measurer (Nano Measurer 1.2.5) software.

In the piezoelectric performance testing, due to the inherent variability in sample preparation, we conducted multiple tests for each data point, using five identical samples per measurement. The best performance result from each group was recorded as the experimental outcome. The β-phase content was calculated using FTIR data from films of the same batch. The analysis followed a standard method, ensuring the accuracy and consistency of the results.

**Figure 2:**
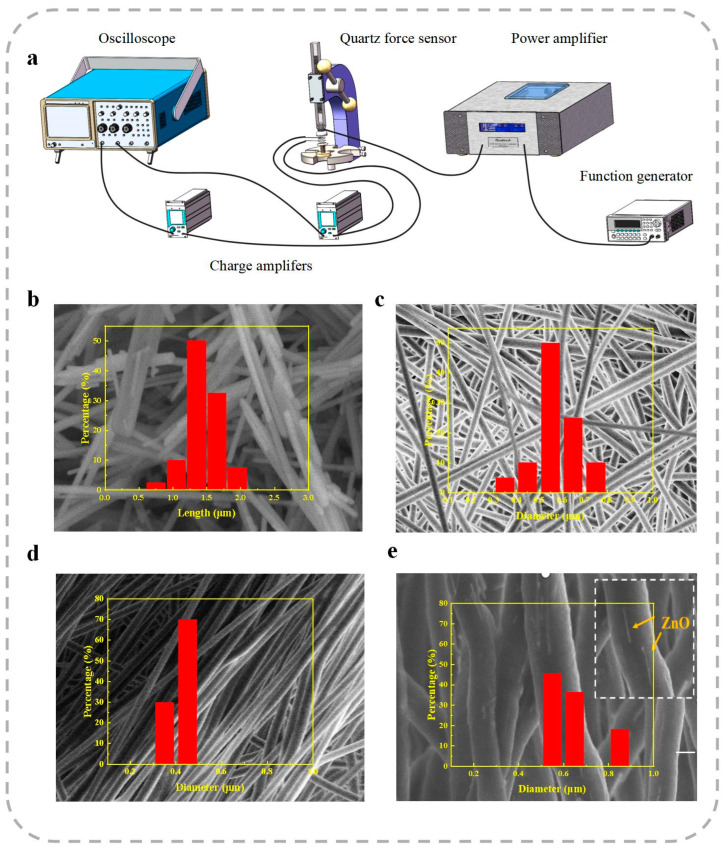
(**a**) Schematic diagram of the self-assembled vertical piezoelectric experimental setup. SEM images of (**b**) ZnO NRs, (**c**) neat P(VDF-TrFE) nanofibers collected at a rotating speed of 300 rpm on the electrospinning drum, (**d**) neat P(VDF-TrFE) nanofibers collected at a rotating speed of 1500 rpm on the electrospinning drum, and (**e**) P(VDF-TrFE) nanofibers doped with ZnO NRs at a mass fraction of 9 wt%, collected at a rotating speed of 1500 rpm on the electrospinning drum.

The electrical measurements, including the open-circuit voltage ($V_{OC}$) and short-circuit current ($I_{SC}$), were recorded using a precision source meter (2612B, Keithley, Cleveland, OH, USA). The vertical piezoelectric performance of the PENGs was evaluated using a custom-designed piezoelectric measurement system, as illustrated in Figure 2a. The piezoelectric signals generated by the nanofiber membrane were captured and recorded using an oscilloscope.

## 3. Results

### 3.1. Microstructures of ZnO NRs

The incorporation of ZnO, in various morphologies, into piezoelectric polymers has shown promise in fabricating biocompatible, high-performance piezoelectric nanofiber membranes. ZnO NRs, in particular, offer notable advantages due to their high aspect ratio and piezoelectric characteristics. Unlike non-piezoelectric high-aspect-ratio fillers, ZnO NRs can synergistically enhance the piezoelectric performance of polymer matrices. Their unique morphology allows for improved mechanical–electrical energy conversion, making them suitable for generating high-performance piezoelectric nanocomposites [42,43].

**Figure 3:**
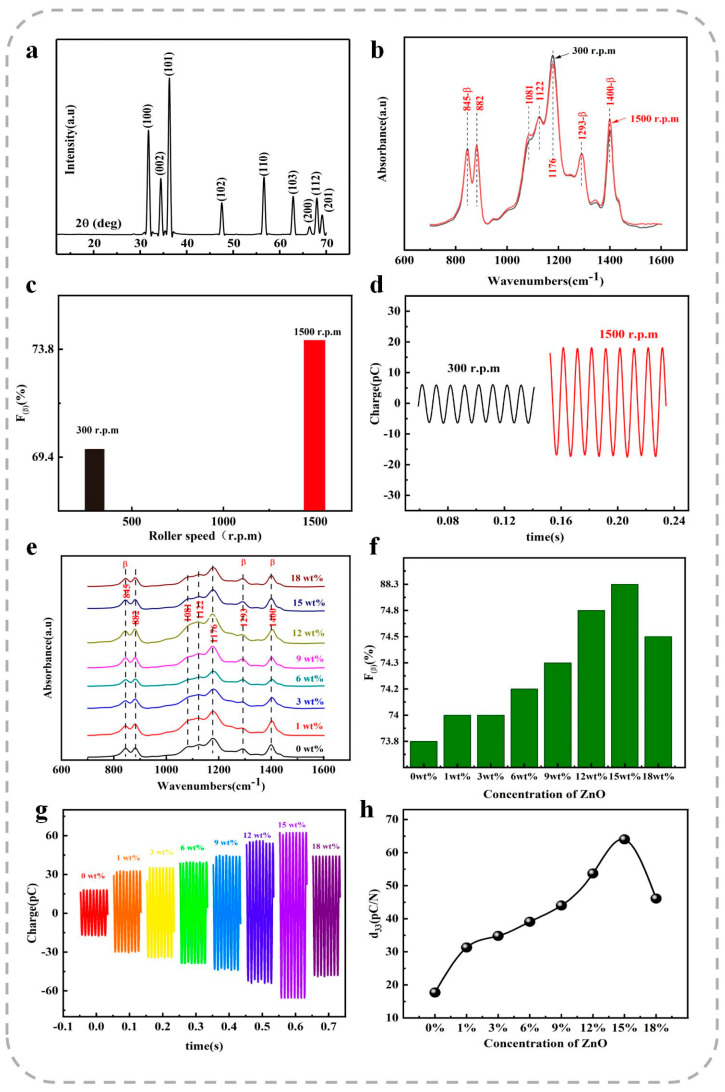
(**a**) XRD patterns of the ZnO NRs. (**b**) FTIR spectra of P(VDF-TrFE) fibers collected at rotating speeds of 300 and 1500 rpm on the electrospinning drum. (**c**) Corresponding β-phase content of P(VDF-TrFE) fibers. (**d**) Induced charges of neat P(VDF-TrFE) films collected at rotating speeds of 300 and 1500 rpm on the electrospinning drum. (**e**) FTIR spectra of P(VDF-TrFE)/ZnO fiber composites with varying doping contents of ZnO NRs. (**f**) Corresponding β-phase content of P(VDF-TrFE)/ZnO fiber composites. (**g**) Induced charges of P(VDF-TrFE) composites with different doping contents of ZnO NRs. (**h**) d₃₃ values of P(VDF-TrFE) composite films with varying doping contents of ZnO NRs.

The morphologies of the ZnO NRs were analyzed using scanning electron microscopy (SEM), as shown in Figure 2b. The as-synthesized ZnO NRs exhibited a uniform morphology with a high aspect ratio. The average length of the ZnO NRs was approximately 1.4 μm, with an average diameter of around 47 nm. The X-ray diffraction (XRD) patterns of the ZnO NRs, presented in Figure 3a, display distinct diffraction peaks corresponding to the hexagonal wurtzite structure of ZnO, which are consistent with the standard data from JCPDS Card No. 75-0576.

### 3.2. Aligned Neat P(VDF-TrFE) Fibers

Electrospinning is a versatile and widely used template-free technique for fabricating piezoelectric polymer fibers. This method enables the production of large-scale, self-polarized piezoelectric polymer nanofiber membranes by applying a high voltage to drive a jet of charged polymer droplets through a metal nozzle. During the fabrication process, polymer nanofibers are formed through mechanical stretching as the charged droplets travel toward a rotating collector. However, this liquid-to-solid transition process often results in randomly distributed fibers, which exhibit suboptimal electrical properties and limited sensitivity to mechanical stimuli. The alignment of electrospun fibers has been demonstrated to significantly enhance their piezoelectric performance. Aligned fibers can be fabricated through post-electrospinning mechanical stretching or by using a collector oriented parallel to the spinning axis during the electrospinning process [44,45,46,47,48]. While these methods improve the alignment of fibers and their piezoelectric properties to some extent, challenges remain in achieving high electrical responses from the resulting fiber membranes. Furthermore, there is a growing demand for the scalable fabrication of biocompatible, high-performance, and well-aligned nanofiber membranes to meet the requirements of advanced applications. Despite ongoing efforts, progress in this area has been limited, highlighting the need for improved strategies to enhance the performance and scalability of aligned piezoelectric nanofiber membranes.

Figure 2c,d depicts neat P(VDF-TrFE) nanofibers collected at rotating speeds of 300 and 1500 rpm on the electrospinning drum, respectively. It can be clearly observed that the nanofibers collected at a rotating speed of 1500 rpm exhibit an oriented arrangement. To further enhance the piezoelectric properties of the fiber films, varying contents of ZnO nanorods (NRs) were used for doping. The doped P(VDF-TrFE) nanofibers were collected at a rotating speed of 1500 rpm on the electrospinning drum. Figure 2e shows a closer examination of the SEM images and reveals that the nanofibers in the region are well-formed, with ZnO NRs aligning along the in-plane direction within the P(VDF-TrFE) polymer matrix, showing good dispersion.

### 3.3. Doped ZnO/P(VDF-TrFE) Fibers

The morphologies of the electrospun ZnO/P(VDF-TrFE) fibers are shown in Figure 2e. It is evident that the ZnO NRs are well-aligned along the axis of the P(VDF-TrFE) fibers and are evenly dispersed within the polymer matrix. Moreover, the interfaces between the ZnO NRs and the P(VDF-TrFE) fibers exhibit strong interfacial bonding, indicating efficient integration of the nanorods into the polymer fibers.

Upon doping P(VDF-TrFE) nanofibers with ZnO NRs, an increase in the fiber diameter was observed. Specifically, the average diameter of the fibers increased from approximately 450 nm for neat fibers to about 600 nm for ZnO doped fibers. This increase in diameter is attributed to the high aspect ratio of the ZnO NRs. During the electrospinning process, the ZnO NRs are aligned along the direction of the P(VDF-TrFE) fibers, which disrupts the typical fiber formation mechanism. The presence of these elongated nanorods facilitates the formation of thicker fibers as the nanorods act as a structural support, preventing the fibers from shrinking to their original dimensions. This interaction between the ZnO NRs and the polymer matrix effectively alters the electrospinning dynamics, leading to an increase in the fiber diameter. Additionally, the incorporation of ZnO NRs into the fiber matrix also influences the overall material structure, contributing to changes in fiber morphology and porosity.

Our findings reveal that increasing the rotational speed of the electrospinning drum to 1500 rpm effectively promotes the overall alignment of the nanofibers [49]. Simultaneously, the incorporated ZnO NRs become oriented along the axis of the nanofibers during the fabrication process. This dual orientation—of both the nanofibers and the ZnO NRs—significantly enhances the piezoelectric performance in both the perpendicular and transverse directions.

## 4. Discussion

### 4.1. Microstructure and Crystallization Behavior of ZnO/P(VDF-TrFE) Nanocomposites

To further examine the effects of the electrospinning drum’s rotating speed and the doping of ZnO nanorods (NRs) on the crystalline structure of P(VDF-TrFE) nanofibers, Fourier transform infrared spectroscopy (FTIR) analysis was performed on both neat P(VDF-TrFE) fibers and ZnO NRs-doped P(VDF-TrFE) fibers. For the neat P(VDF-TrFE) fibers collected at different rotating speeds of the electrospinning drum, multiple characteristic peaks were observed, as shown in Figure 3a. The infrared spectra revealed vibrational bands in the frequency range of 700–1600 cm⁻^1^. Three characteristic peaks corresponding to the β-crystalline phase were identified at 845 cm⁻^1^, 1293 cm⁻^1^, and 1400 cm⁻^1^. Specifically, the absorption peaks at 845 cm⁻^1^ and 1293 cm⁻^1^ were attributed to the symmetric stretching vibrations of -CF_2_ and C–C, respectively, while the peak at 1400 cm⁻^1^ was associated with -CH_2_ oscillations and the antisymmetric stretching of C–C. Notably, no significant absorption peak corresponding to the α-crystalline phase was detected at 760 cm⁻^1^, indicating that the electrospun fiber membrane predominantly consists of the β-crystalline phase.

When the rotating speed of the electrospinning drum was increased from 300 rpm to 1500 rpm, the collected nanofibers exhibited a transition from a random arrangement to an oriented alignment, as illustrated in Figure 2c,d. Additionally, the average diameter of the nanofibers decreased from 0.55 μm to 0.45 μm. This transition can be attributed to the enhanced mechanical stretching exerted by the higher rotating speed of the electrospinning drum. As shown in Figure 3b, the absorption peak at 1400 cm⁻^1^ became more prominent, while the peak at 1176 cm⁻^1^ was significantly diminished. These findings indicate a gradual transformation of the non-polar phase in P(VDF-TrFE) into the electroactive β-phase. The axial stretching of the fibers during the electrospinning process facilitates the alignment of polar molecules in the polymer chains at specific angles, thereby increasing polarization and enhancing the β-crystalline phase content.

To precisely evaluate the influence of the rotating speed of the electrospinning drum on the crystalline structure of P(VDF-TrFE), the content of the electroactive β-phase was calculated using the infrared spectral data and Equation (1):

(1) $$F_{\beta }=\frac{A_{\beta }}{\left[(\frac{K_{\beta }}{K_{\alpha }})A_{\alpha }+A_{\beta }\right]},$$

where $A_{\alpha }$ and $A_{\beta }$ represent the absorbance of the α-phase and β-phase at 766 cm⁻^1^ and 840 cm⁻^1^, respectively, and $K_{\alpha }$ and $K_{\beta }$ are the absorption coefficients of the α-phase and β-phase, with values of $6.1\times 10^{4}$ and $7.7\times 10^{4}$ cm^2^/mol, respectively. As shown in Figure 3c, the β-phase content of the fibers collected at a rotating speed of 1500 rpm was 73.8%, compared to 69.4% for the fibers collected at 300 rpm. This result indicates that the oriented fiber films exhibit higher crystallinity than their disordered counterparts due to increased molecular alignment induced by the higher rotating speed of the drum.

The β-phase content was further calculated for P(VDF-TrFE) nanofibers doped with varying concentrations of ZnO NRs and collected using the high-speed electrospinning drum, as presented in Figure 3f. The results demonstrate that the incorporation of ZnO NRs significantly enhances the β-phase content of the P(VDF-TrFE) nanofibers. A maximum β-phase content of 88.3% was achieved at a doping concentration of 15 wt% ZnO NRs. The initial increase in β-phase content with increasing ZnO NR doping can be attributed to the stronger intermolecular interactions exerted by the ZnO NRs, which promote molecular alignment of the P(VDF-TrFE) polymer chains and improve crystallinity. However, when the ZnO NR doping concentration reached 18 wt%, a noticeable decline in β-phase content was observed. This reduction is likely due to the dominant positive piezoelectric effect of ZnO NRs, which counteracts part of the negative piezoelectric effect inherent to P(VDF-TrFE). This counteraction diminishes the overall piezoelectric performance of the composite film, thereby reducing crystallinity and electroactivity in the fiber membrane.

This observation highlights the critical importance of optimizing the ZnO NR doping concentration to achieve a balance between the alignment forces induced by ZnO NRs and the structural integrity of the polymer matrix. Such optimization is essential for maximizing the piezoelectric performance and preserving the crystalline properties of the composite nanofibers.

### 4.2. Electromechanical Conversion Performance of ZnO/P(VDF-TrFE) Nanocomposites

Piezoelectric materials, owing to their mechanical-to-electrical energy conversion characteristics, hold significant potential for applications in energy harvesting, pressure sensing, and bio-sensing. To effectively serve these purposes, it is essential for piezoelectric materials to exhibit excellent piezoelectric properties. In this study, PENGs were fabricated using a simple sandwich structure, as illustrated in Figure 1c, to facilitate the measurement of their piezoelectric performance.

The vertical piezoelectric characteristics were evaluated by applying mechanical excitation along the thickness direction of the film and recording the resulting electrical response along the same direction. Conversely, the transverse piezoelectric characteristics were assessed by applying mechanical stress along the surface direction of the film and measuring the corresponding electrical response along the film’s thickness direction.

The excitation frequency of 100 Hz was chosen based on prior research which showed that a variation in frequency (between 100, 200, and 300 Hz) did not significantly affect the piezoelectric performance of the material. Therefore, 100 Hz was selected to ensure that the results were consistent and did not introduce unnecessary complexity. This frequency is likely optimal for capturing the material’s response under typical conditions.

It is important to note that all measurements were conducted under relatively low applied strain levels, which were insufficient to induce any observable plastic deformation in the material. This ensured that the observed electrical responses were attributed solely to the piezoelectric behavior of the material, rather than any structural changes or damage. These results provide a clear understanding of the piezoelectric performance of the fabricated PENGs and their suitability for various applications requiring efficient mechanical-to-electrical energy conversion.

The 2 N amplitude was chosen as it provided the most stable output for the material’s piezoelectric performance. In previous tests, it was observed that at this level of excitation force, the sample produced the most reliable and consistent piezoelectric output. The elastic modulus of the sample also played a role here, as the material’s deformation behavior under this force led to the most stable measurements, making it an ideal choice for this experiment.

The vertical piezoelectric properties of the PENGs were evaluated using a custom-designed piezoelectric performance testing system. This system comprised a vibrator, a power amplifier, a function generator, a commercial quartz force sensor, and a charge amplifier, as illustrated in Figure 2a. Both the PENG and the quartz force sensor were placed on the vibrator. The vibrator was driven by a voltage signal generated by the function generator, which was pre-amplified using the power amplifier. By adjusting the amplitude, frequency, and waveform of the excitation voltage, mechanical forces with specific characteristics could be applied to the nanofiber membranes and the quartz sensor.

The induced charges generated in the P(VDF-TrFE) nanofiber membrane under a sinusoidal mechanical excitation frequency of 100 Hz and an amplitude of 2 N are presented in Figure 3d. For the aligned neat P(VDF-TrFE) fiber film collected at an electrospinning drum rotating speed of 1500 rpm, the induced charge was measured to be 35.4 pC, which is 187.8% higher than that of the randomly distributed fibers collected at a drum rotating speed of 300 rpm. This improvement can be attributed to the increased mechanical stretching forces exerted by the high-speed electrospinning drum, which enhance the alignment of the fibers. Additionally, the higher degree of alignment leads to improved crystallinity of the nanofiber films, thereby increasing their piezoelectric responses.

For composite films with different doping contents of ZnO NRs (0–18 wt%) collected at a drum rotating speed of 1500 rpm, the maximum induced charge was observed at a doping content of 15 wt%. At this doping level, the induced charge reached a peak value of 124.8 pC under a cyclic pressure of 2 N and a frequency of 100 Hz. Correspondingly, the $d_{33}$ value was calculated to be −62.4 pC/N, which is 252.5% higher than that of PENGs fabricated from neat P(VDF-TrFE). This $d_{33}$ value also exceeds the values reported in previous studies ($d_{33}$), ranging from −21.0 to −52.0 pC/N [50,51,52,53,54]. Figure 3g,h illustrates the induced charges and the $d_{33}$ values of the composite films with varying ZnO NR doping contents, respectively.

The doping range of 0–18 wt% was selected based on observed trends in our experiments where increasing the doping concentration of ZnO NRs initially enhanced piezoelectric performance but after a certain threshold, the performance began to decline. At 0–18 wt%, both the transverse and longitudinal piezoelectric performance showed a clear increase and subsequent decrease, allowing for a full characterization of the doping effect. Beyond 18 wt%, the piezoelectric properties were found to worsen, hence the range was capped at 18 wt% for this study.

A comparison between Figure 3f and Figure 3g reveals that the trend in β-phase content aligns closely with the trend in induced charges as the doping content of ZnO NRs varies. This suggests that the piezoelectric properties of the polymer in the vertical piezoelectric test are predominantly influenced by the β-phase content. The enhanced β-phase content in the composite films with optimized ZnO NR doping contributes significantly to their superior piezoelectric performance.

The transverse piezoelectric properties are particularly critical for PENGs in specific application scenarios, such as wearable sensors for human motion gesture recognition, arterial pulse monitoring, and eye fatigue detection. The experimental setup for transverse piezoelectric characterization is schematically illustrated in Figure 3a. In this system, the nanofiber membranes are fixed onto a linear motor, and the movement of the motor induces compression and stretching of the membranes. The resulting electrical responses are recorded using a source meter. The strain applied to the membranes and the frequency (f) of the bending–releasing process are controlled by adjusting the final displacement (Δd) and the speed (v) of the motor’s slider.

During the measurements, the bottom electrode of the nanofiber membrane is electrically grounded, and the nanofibers are bent upward to perform the transverse piezoelectric tests. Additionally, the test specimen was straightened before initiating the bending operation. The initial distance ($d_{0}$) between the slider and the fixed end of the setup is set to 35 mm.

The difference in optimal ZnO concentrations for the transverse and vertical piezoelectric responses is a complex phenomenon related to the anisotropic nature of the composite material. The alignment of the ZnO nanoparticles within the polymer matrix can vary depending on the direction of the applied stress. In the vertical configuration, where the stress is applied perpendicular to the film, the alignment of the particles may differ from that in the transverse configuration, where the stress is applied parallel to the surface. As a result, the ZnO nanoparticles may interact differently with the polymer matrix in these two orientations, leading to variations in the optimal concentration for each type of response. This behavior is influenced by both the mechanical properties of the matrix and the piezoelectric properties of the ZnO nanoparticles themselves.

**Figure 4:**
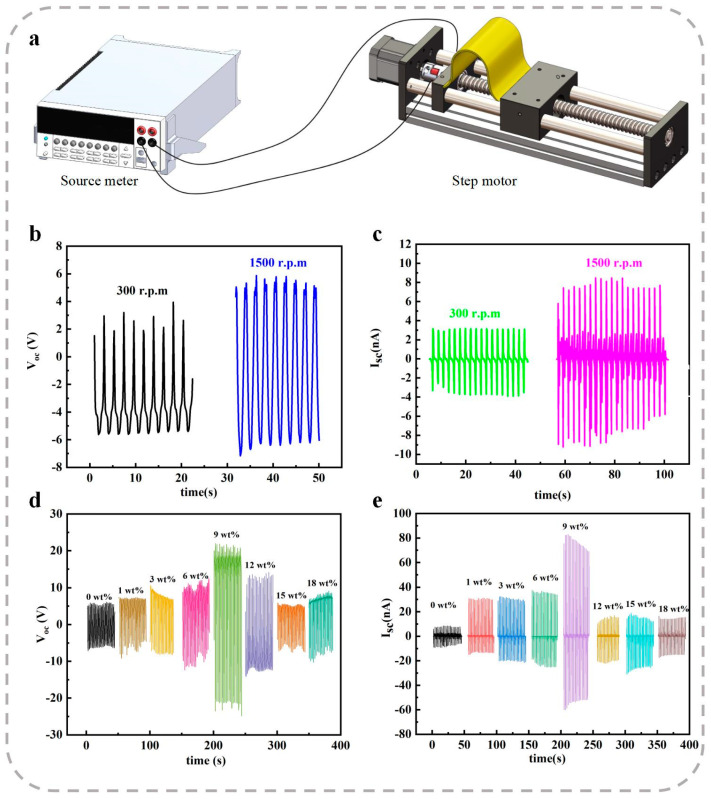
Measurement of transverse piezoelectric properties. (**a**) Schematic diagram of the measurement system. (**b**) $V_{OC}$ and (**c**) $I_{SC}$ of neat P(VDF-TrFE) fiber films collected at electrospinning drum rotating speeds of 300 rpm and 1500 rpm. (**d**) $V_{OC}$ and (**e**) $I_{SC}$ of P(VDF-TrFE) composite films with varying ZnO NR doping contents.

The typical $V_{OC}$ responses of all membranes under a slider speed of v = 9.5 mm/s and a displacement of d = 20 mm are shown in Figure 4b,d. A negative $V_{OC}$ output is observed during the bending half-cycle, while a positive $V_{OC}$ response is detected during the releasing half-cycle. These alternating outputs confirm the piezoelectric behavior of the membranes under transverse mechanical deformation. Furthermore, the $I_{SC}$ outputs of the composite membranes are presented in Figure 4c,e. The consistent $I_{SC}$ signals further demonstrate the effective transverse piezoelectric response of the membranes.

These results highlight the potential of the P(VDF-TrFE)-based nanofiber membranes, with or without ZnO NR doping, for applications requiring transverse piezoelectric energy conversion or sensing, particularly in wearable and flexible electronic devices.

As presented in Figure 4b,c, the transverse piezoelectric properties of the fiber films were investigated to further evaluate the effect of increasing the rotation speed of the electrospinning drum. The fiber films collected at a drum rotating speed of 1500 rpm demonstrated superior electrical output performance compared to those collected at lower speeds. Specifically, the $V_{OC}$ and $I_{SC}$ of the neat P(VDF-TrFE) fiber film collected at 1500 rpm reached 12.0 V and 16.7 nA, respectively. In contrast, the $V_{OC}$ and $I_{SC}$ of the P(VDF-TrFE) fiber film prepared at a lower drum rotating speed of 300 rpm were 8.8 V and 6.8 nA, respectively. These results indicate that the use of a high-speed electrospinning drum effectively enhances the transverse piezoelectric properties of the fiber films due to improved fiber alignment and crystallinity.

The transverse piezoelectric properties of composite fiber films doped with varying mass fractions of ZnO nanorods (NRs) and prepared at a drum rotating speed of 1500 rpm are shown in Figure 4d,e. As the ZnO NR doping content increased, the $V_{OC}$ and $I_{SC}$ of the composite films initially increased and subsequently decreased. The maximum $V_{OC}$ and $I_{SC}$ were observed at a ZnO NR doping content of 9 wt%, reaching 42.6 V and 138.8 nA, respectively. These values represent significant enhancements of 255.0% and 731.1%, respectively, compared to the neat P(VDF-TrFE) fiber film.

The enhanced performance observed at the optimal ZnO NR doping level can be attributed to the incorporation of ZnO NRs into the P(VDF-TrFE) matrix, which promotes an increase in the electroactive β-phase content and enhances the overall crystallinity of the fiber membrane. This improvement leads to a higher proportion of the effective piezoelectric β-phase, a critical factor in improving piezoelectric performance.

As ZnO NRs are introduced into the P(VDF-TrFE) matrix, they could influence the polymer’s crystallization behavior by altering the local electrostatic fields or the molecular orientation of the polymer chains. This can be used to explain the initial increasing of the β-phase. In the case of PDMS-based composites, the reduction in piezoelectric response has indeed been linked to the hardening of the polymer matrix [55], which restricts the mobility of dipoles and limits the piezoelectric effect. In our study, while there is no β-phase in the ZnO itself, the interaction between the ZnO nanoparticles and the polymer may result in a similar phenomenon, specifically as the concentration of ZnO increases. This hardening effect could explain the reduction in β-phase at higher ZnO concentrations, similar to what is observed in PDMS-based composites, leading to its reduction at higher concentrations of ZnO NRs. Similarly, a decline in piezoelectric performance is observed at higher ZnO NR doping levels. This phenomenon can be explained by the positive piezoelectric effect of ZnO NRs, which counteracts the intrinsic negative piezoelectric effect of P(VDF-TrFE). This counteraction reduces the overall piezoelectric response of the composite film.

As a result, the piezoelectric performance of the composite fiber membranes decreases when the ZnO NR doping content exceeds the optimal level. These results highlight the necessity of optimizing ZnO NR doping content to achieve superior transverse piezoelectric properties in P(VDF-TrFE)-based fiber films. This optimization is essential for maximizing the piezoelectric output and ensuring the effective integration of ZnO NRs into the P(VDF-TrFE) matrix.

**Figure 5:**
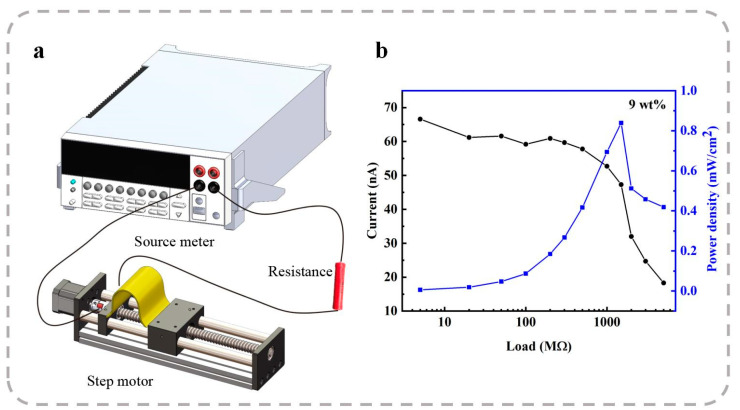
Mechanical energy harvesting from nanofiber membranes. (**a**) Schematic diagram of the measurement system used for mechanical energy harvesting. (**b**) Peak current outputs and the corresponding peak power density values as a function of the external resistance load.

The piezoelectric nanofiber membrane capacitor was further optimized for mechanical energy harvesting applications. As illustrated in Figure 5a, the energy harvester was electrically connected in parallel with a variable resistor to evaluate the optimal external load and determine the maximum power density generated. A source meter was employed to measure the current drop across the variable resistor, which was induced by the movement of a linear motor.

**Figure 6:**
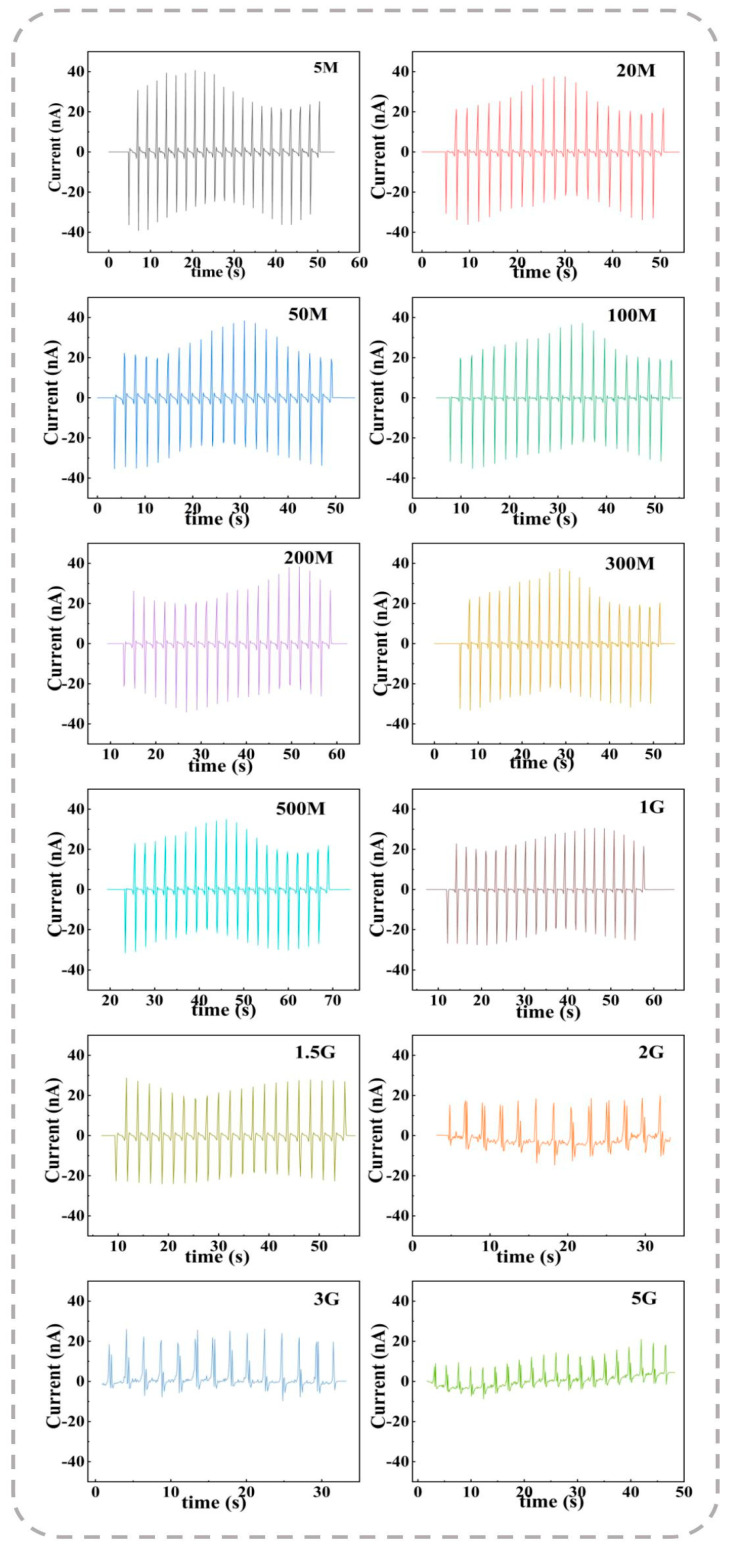
Current responses of 9 wt% ZnO-doped P(VDF-TrFE) membranes under repeated bending operations with varying external resistance loads.

Figure 6 presents the current responses of the 9 wt% ZnO-doped nanofiber membrane under different external resistance loads ($R_{var}$). The results indicate that the current response increases proportionally with the external resistance for the tested nanofiber membranes. To further analyze the output performance, the peak current outputs ($I_{peak}$) were extracted from Figure 6 and plotted as a function of the external resistance in Figure 5b. The corresponding peak electrical power density ($P_{peak}$) generated per unit area for each external resistance load was calculated using Equation (2):

(2) $$P_{peak}=\frac{I_{peak}^{2}R_{\mathrm{var}}}{A},$$

where A represents the effective electrode area (4 cm^2^). The peak power density ($P_{peak}$) for each nanogenerator initially increases as the external resistance ($R_{var}$) rises from 5 MΩ to 1.5 GΩ, and subsequently decreases beyond this value. As a result, an optimal external resistance of 1.5 GΩ yields a maximum $P_{peak}$ value of 0.84 μW/cm^2^ for the 9 wt% ZnO-doped P(VDF-TrFE) membranes. This achieved maximum power density is notably higher than the 0.53 μW/cm^2^ reported for BaSrTiO_3_-doped P(VDF-TrFE) membranes [56]. These findings highlight the enhanced energy harvesting performance of ZnO-doped P(VDF-TrFE) membranes over other doped systems, making them promising candidates for high-performance piezoelectric energy harvesters.

**Figure 7:**
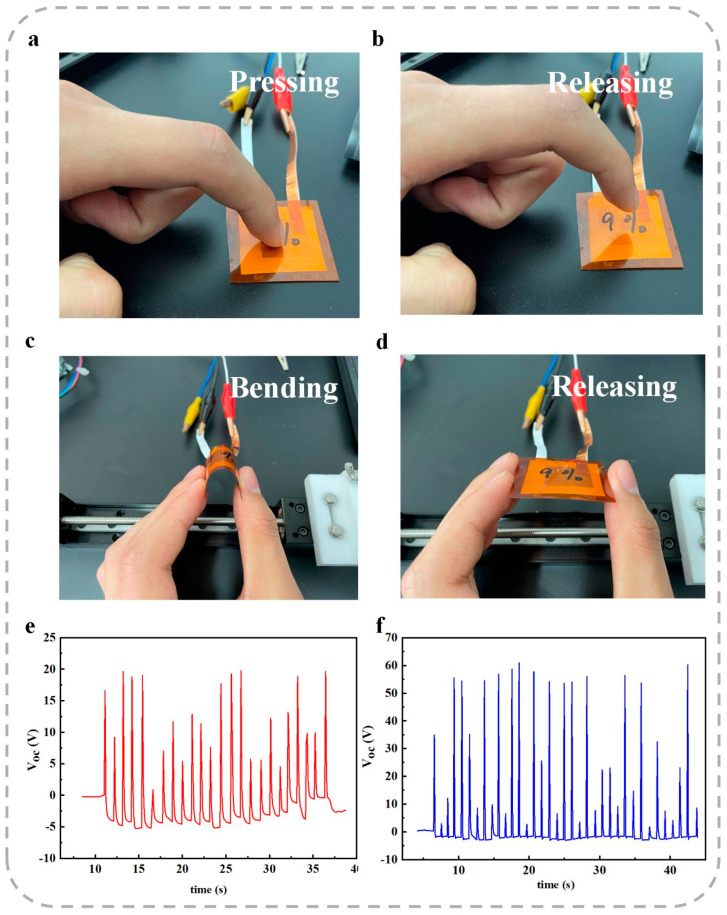
Application of the 9 wt% ZnO-doped nanofiber membrane as a wearable sensor for the detection of (**a**,**b**) finger tapping and (**c**,**d**) bending and releasing of the sensor. (**e**) $V_{OC}$ response to finger tapping. (**f**) $V_{OC}$ response to bending and releasing of the sensor.

Finally, to evaluate the potential application of the ZnO-doped nanofiber membrane as a wearable sensor, a series of tests involving finger tapping, bending, and releasing were conducted. As shown in Figure 7, the sensor exhibited distinct $V_{OC}$ responses of approximately 20 V for finger tapping and 60 V for bending and releasing.

These results demonstrate the significant sensitivity and responsiveness of the sensor, suggesting its strong potential for further development in various applications, particularly in the field of human physiological monitoring. This highlights the versatility and practicality of the ZnO-doped nanofiber membrane for wearable sensing technologies.

## 5. Conclusions

In this study, highly aligned P(VDF-TrFE) nanofiber membranes were successfully fabricated using the electrospinning technique. The incorporation of ZnO nanorods (NRs) into the fiber membranes resulted in preferential alignment of the ZnO NRs along the fiber direction, enhancing the overall structural and piezoelectric properties of the membranes.

The alignment of the P(VDF-TrFE) nanofibers significantly improved their piezoelectric performance. The $d_{33}$ of the aligned fibers increased by 187.8% compared to randomly oriented fibers. In transverse measurements, the $V_{OC}$ and $I_{SC}$ of the aligned membranes exhibited enhancements of 36.4% and 145.6%, respectively.

The addition of ZnO further augmented the piezoelectric properties. At a ZnO doping level of 15 wt%, the vertical $d_{33}$ value increased by 252.5%, while the transverse $V_{OC}$ and $I_{SC}$ exhibited dramatic improvements of 255.0% and 731.1%, respectively, at a doping content of 9 wt%.

Moreover, the optimized nanofiber membranes demonstrated a peak power density of 0.84 μW/cm^2^ at an optimal load resistance, confirming their potential for energy harvesting applications. Additionally, the ZnO-doped P(VDF-TrFE) nanofiber membranes were successfully applied as wearable sensors, effectively detecting finger tapping, bending, and releasing motions, with corresponding $V_{OC}$ responses of approximately 20 V and 60 V.

These results highlight the significant enhancement of piezoelectric and power generation properties in ZnO-doped P(VDF-TrFE) nanofiber membranes. The findings validate the potential of these materials for advanced applications in wearable and flexible electronics, paving the way for future developments in this field.

There are several avenues for potential future work that could further enhance the practical applications of this technology:

Optimization of ZnO Doping Levels: While this study explored doping at various concentrations, further research could focus on fine-tuning the optimal doping levels for different applications, balancing performance with material stability over long-term use.

Scalability and Fabrication Methods: Scaling up the electrospinning process for large-area, high-throughput production while maintaining the alignment of nanofibers could be a critical next step. This would help in addressing the manufacturing challenges associated with deploying these materials in mass-market wearable and flexible devices.

Human-Body Interface and Health Monitoring: Further studies could focus on expanding the range of human physiological signals that can be captured by these sensors, such as muscle movement, heart rate, or even respiration patterns. Incorporating additional sensor modalities could lead to more comprehensive health monitoring solutions.

By pursuing these research directions, future work could lead to the refinement and broader adoption of ZnO-doped P(VDF-TrFE) nanofiber membranes in advanced wearable and flexible electronics, making them more efficient, versatile, and reliable for a range of applications in healthcare and energy harvesting.

## Data Availability

All the data of this study are included in the manuscript.

## Abbreviations

P(VDF-TrFE)	Poly(vinylidene fluoride-trifluoroethylene)
ZnO	Zinc oxide
NRs	Nanorods
LD	Linear dichroism
PENGs	Piezoelectric nanogenerators
PVDF	Poly(vinylidene fluoride)
TGTG	Trans–gauche–trans–gauche configuration
TTTT	All-trans configuration
PZT	Lead zirconate titanate
BTO	Barium titanate
Zn(CH_3_COO)_2_∙2H_2_O	Zinc acetate dihydrate
DMF	N, N-dimethylformamide
NaOH	Sodium hydroxide
FTIR	Fourier transform infrared spectra
$V_{OC}$	Open-circuit voltage
$I_{SC}$	Short-circuit current
$d_{33}$	Vertical piezoelectric coefficient
$I_{peak}$	Peak current outputs
$P_{peak}$	Peak power density
$R_{var}$	External resistance
